# Sex-specific clinical profiles and long-term outcomes in adolescents and young people at clinical high risk for psychosis: Findings from the PARMS program

**DOI:** 10.1007/s00787-026-03082-8

**Published:** 2026-06-04

**Authors:** Lorenzo Pelizza, Camilla Ricci, Emanuela Leuci, Emanuela Quattrone, Derna Palmisano, Simona Pupo, Giuseppina Paulillo, Clara Pellegrini, Pietro Pellegrini, Marco Menchetti

**Affiliations:** 1 Department of Biomedical and Neuromotor Sciences, Alma Mater Studiorum – Università di Bologna, Bologna (BO), Italy; 2 Department of Mental Health and Pathological Addiction, Azienda USL di Parma, Parma, PR Italy; 3 Pain Therapy Service, Department of Medicine and Surgery, Azienda Ospedaliero-Universitaria di Parma, Parma, PR Italy; 4 Istituto di Psichiatria “Paolo Ottonello” - via Pepoli, Bologna (BO), 5 - 40126 Italy

**Keywords:** Prodrome, Early psychosis, Sex Differences, Early Intervention, Outcome, Follow-up

## Abstract

Males with first episode psychosis (FEP) show poorer premorbid functioning, more severe psychopathology, and lower functional remission than females. Preliminary evidence suggests that even in Clinical High-Risk for Psychosis (CHR-P), males and females differ in clinical and psychosocial profiles. However, further investigations are needed, especially about outcomes and in adolescents. This study aimed to examine sex differences in clinical/functional presentation and longitudinal outcomes between CHR-P men and women assessing the impact of specialized “Early Intervention in Psychosis” (EIP) interventions over a two-year follow-up within an Italian program. 179 CHR-P men and women (90 females, 89 males; aged 12–25 years) were assessed using the Health of the Nation Outcome Scale and the Positive and Negative Syndrome Scale at baseline and annually for two years. Of them, 152 (76 males, 76 females) completed the follow-up. At baseline, males were older, had higher rates of substance use and unemployment, more severe negative symptoms, and poorer insight. They frequently suffered from brief limited, intermittent psychotic symptoms, whereas females predominantly from attenuated psychotic symptoms. Over two years, females showed a significantly higher rate of functional remission. Specialist EIP interventions increased across time and potentially contributed to improve outcomes in both sexes. Sex differences are evident at baseline in CHR-P participants, especially regarding psychopathology and social functioning. Nonetheless, clinical trajectories over time are largely comparable, except for greater functional recovery in females. These findings support the time effects of our early interventions regardless of sex but emphasize to further explore sex-specific pathways in psychosis risk and recovery.

**Supplementary Information:**

The online version contains supplementary material available at https://doi.org/10.1007/s00787-026-03082-8.

## Introduction

The role of *sex* in determining the course and prognosis of psychosis has been extensively studied in the literature, with evidence of differences in psychopathology, clinical presentation and longitudinal trajectories between men and women [1–5]. Despite the growing interest in early detection/intervention in psychosis, relatively few studies have specifically examined sex differences among individuals at “Clinical High Risk for Psychosis” (*CHR-P*). Some evidence suggest that males and females exhibit distinct clinical and psychosocial profiles even in the early stages of psychosis risk. According to a comprehensive review by Barajas and co-authors [6], males with CHR-P at baseline tend to exhibit more severe negative symptoms, poorer social functioning and a longer “Duration of Untreated Illness” (DUI) compared to females. These findings overall confirm previous results reported by Willhite and colleagues [7], who observed higher negative symptom severity and lower functional outcomes in CHR-P males, whereas CHR-P females showed stronger perceived social support, which could act as a protective factor.

Other authors suggested that sex differences may play a role in psychosis transition. In this respect, Fusar-Poli and co-authors [8] found that also sociodemographic and environmental factors could compete as risk factors for the transition to psychosis, such as single status, unemployment, low educational or functional level, obstetric complications, childhood trauma and male sex. However, CHR-P individuals often have several risk factors, and this could explain the higher rate of psychosis transition in males compared to females. In this regard, a meta-analysis by He and colleagues [9] reported a 22.3% psychosis transition rate in males at CHR-P and a 20.4% rate in females, with a statistically significant higher transition risk in CHR-P men.

Furthermore, Chan and co-authors [10] recently found that CHR-P males exhibited poorer social functioning and more severe asociality at baseline than females. No other differences were observed in other symptom domains, cognitive measures, and role functioning, suggesting that sex differences in CHR-P people may be less pronounced than those observed in established psychotic disorders. As for cognition, a study from the European Gene-Environment Interactions (EUGEI) group showed that women performed better in speed of processing and verbal learning compared to men, regardless of whether they were CHR-P or HC, suggesting that differences in cognition may be not specific for CHR-P [11]. Furthermore, men with first episode psychosis (FEP) had worse premorbid academic adjustment [12] and, overall, showed a greater deterioration in their premorbid functioning, which also predicts a longer duration of untreated psychosis [13]. This sex-based worsening in early academic functioning has also been replicated in HC [12].

Despite these interesting observations, evidence on sex differences in CHR-P are still scarce and not definitive, especially regarding their impact on outcomes and treatment response. For this reason, the main *aims* of this study were: (a) to examine any difference in baseline clinical and functioning presentation between CHR-P males and females treated within an Italian Early Intervention in Psychosis (EIP) program, and (b) to longitudinally investigate post-intervention outcome differences by sex and the potential beneficial effects of specialized psychosocial and pharmacological treatments provided to the CHR-P total group, specifically focusing on a broad range of clinical and functional outcomes across a 2-year follow-up period.

## Methods

### Sample and setting

CHR-P participants were consecutively recruited within the Parma At-Risk Mental States (PARMS) program from 1 st January 2016 to 31 st December 2022. The *PARMS* protocol is a specialized EIP infrastructure diffusely implemented across all adult and adolescent mental health services in the Parma Department of Mental Health (Northern Italy) [14].

*Inclusion criteria* were: (a) specialist mental health request; (b) age 12–25 years, (c) to meet CHR-P criteria as defined by the “Comprehensive Assessment of At-Risk Mental States” (CAARMS) [15] at the baseline assessment (i.e., “Genetic Vulnerability” (GV), “Attenuated Psychotic Symptoms” [APS], or “Brief Limited Intermittent Psychotic Symptoms” [BLIPS]). This age range was specifically selected because it is the usual limit considered in CHR-P intervention programs [16].

*Exclusion criteria* were: (a) previous overt (affective or non-affective) psychotic episode; (b) past exposure to antipsychotic (AP) medication or current AP intake exceeding 4 weeks in the present episode, (c) known intellectual disability (IQ < 70); and (d) neurological or medical disorder with psychiatric manifestations. Previous use of AP drug was considered a proxy for a past psychotic episode, consistently with the original CAARMS criteria for psychosis threshold [17]. A current AP prescription of less than 4 weeks was also required to minimize pharmacological interference with baseline psychopathological assessment [18]. Intellectual disability was assessed using the “Wechsler Adult Intelligence Scale, IV edition” (WAIS-IV) [19] or the “Wechsler Intelligence Scale for Children, IV edition (WISC-IV) [20] as appropriate.

### Instruments

The psychopathological assessment included the CAARMS, the “Health of the Nation Outcome Scale” (HoNOS) [21], the “Positive And Negative Syndrome Scale” (PANSS) [22], and the “Social and Occupational Functioning Assessment Scale” (SOFAS) [23].

The *CAARMS* is a clinical interview designed to explore various aspects of attenuated psychopathology. Its “Positive Symptoms” subscale was used for defining CHR-P criteria. CAARMS interviews were conducted by trained PARMS team members using the approved Italian version [24]. Regular CAARMS scoring workshops and supervision sessions were implemented to ensure good inter-rater reliability values [25]. Every 12 months in the follow-up, CAARMS interview was re-administered to psychometrically identify psychosis transition and CHR-P criteria persistence.

The *HoNOS* was developed to evaluate mental health and social functioning of individuals with severe mental illness, including patients at CHR-P [21]. As originally indicated [26], subscale scores were derived by grouping items into four main domains: “Behavioral Problems”, “Impairment”, “Psychiatric Symptoms”, and “Social Problems”. According to Kortrijk and co-authors [27], we considered a score of ≤ 2 on the HoNOS items 9, 10 and 11 as index of *functional remission*. In the present research, we used the Italian version of the HoNOS, which showed good psychometric properties in Italian patients with severe mental illness [28].

The *PANSS* is a widely used clinical interview for assessing psychopathology in psychosis, including its early phase [29, 30]. As indicated by Shafer and Dazzi [31], we considered five principal psychopathological factors: “Disorganization”, “Negative Symptoms”, “Positive Symptoms”, “Resistance/Excitement-Activity”, and “Affect”. As index of *symptomatic remission*, we considered a score of ≤ 3 (corresponding to mild severity or less) on the 8 PANSS items indicated by the “Remission in Schizophrenia Working Group’s criteria” (see also Supplementary Materials [Table S1] for details) [32].

The *SOFAS* is a commonly used scale for evaluating socio-occupational functioning in CHR-P individuals and is included in the CAARMS. According to Schennach-Wolff and colleagues [33], we considered a SOFAS score of > 61 at follow-ups as current index of *functional remission*. Therefore, based on current research on CHR-P conditions, two different but complementary indices, previously used, were calculated to define functional remission: one based on the SOFAS score (which reflects a composite measure of socio-occupational functioning) and the other based on the HoNOS scores (which assess specific items describing problems with interpersonal relationships, activity of daily living, and housing/living conditions). Finally, as proposed by Silva and Restrepo [34], we also used another practical index of “functional recovery”, simply defined as “return to work or school”. This versatile choice was primarily intended to make the broadest possible comparisons on functional remission with other comparable CHR-P studies. However, no specific assessment was conducted to measure premorbid adjustment/functioning.

Additionally, we completed a sociodemographic and clinical/treatment chart, including a wide range of information (such as age at entry, years of education, sex, civil status, living situation, ethnic group and migration status, substance abuse at presentation, source of referral, past hospitalization, previous specialist contact, and PARMS treatment component). Specifically, the term “Occupation” indicated a job or profession, including the status of student. Furthermore, according to the current literature, participants who were Not engaged in Education, Employment or Training were grouped in the “NEET” subgroup [35]. In this sense, occupation was assessed separately through NEET/student/employment status. DUI was defined as the time interval between the first psychiatric manifestation and the first pharmacological/psychosocial intervention [36]. Suicide attempt was defined as potentially injurious, self-inflicted behavior without a fatal outcome for which there was (implicit or explicit) evidence of intent to die, while self-harm behaviors were acts of deliberate self-harm or intoxication with alcohol or drugs, but where there was no clear intention to die [37]. Service disengagement was defined as a complete lack of contact or untraceable for at least 3 months despite a need for treatment [38]. Current suicidal ideation was detected as a score of ≥ 2 on item 4 (“Suicidality”) of the Brief Psychiatric Rating Scale (BPRS), corresponding to “at least to occasional suicidal thinking without specific plans” [39].

All assessment tools were administered both at baseline (T0) and every 12 months during the 2-year follow-up period (i.e., T1 and T2). Finally, the initial *DSM-5 diagnosis* was formulated at entry by a minimum of two trained PARMS team members using the “Structured Clinical Interview for DSM-5 mental disorders” (SCID-5) [40].

### Procedures

After the initial DSM-5 diagnosis was made, CHR-P participants were divided into two subgroups based on sex. The term “sex” was used to refer to a binary of being either male or female as denoted by attributes that comprise biological sex [41]. Between-group comparisons on sociodemographic, clinical and treatment measures at baseline were first investigated. The two subsamples were then examined for clinical/psychopathological outcomes across the 2-year follow-up period. Specifically, longitudinal incidence rates of a broad range of relevant outcome parameters (i.e., service disengagement, new hospitalization, new suicide attempt, psychosis transition) were collected each 12 months during the follow-up. Lastly, inter-group comparisons on treatment response were also investigated.

Within 3–4 weeks from the PARMS enrollment, CHR-P individuals were assigned to a *multi-professional team* (including a clinical psychologist, an early rehabilitation case manager, and a psychiatrist) offering specialized psychopharmacological and psychosocial treatments [42]. The duration of this EIP intervention was 2 years and was similar between groups. Specifically, psychosocial interventions included individual psychotherapy, family psychoeducation, and dedicated case-management (when necessary) to support and promote early recovery-oriented rehabilitation (see Supplementary Materials [Table S2] for details).

### Statistical analysis

Collected data were analyzed using the Statistical Package for Social Science (SPSS) 15.0 for Windows [43]. All tests were two-tailed, with a significance level set at 0.05. Where applicable, Bonferroni’s p correction was applied to reduce the statistical impact of multiple comparisons [44]. Given non-normality distributions of parameters at baseline (Kolmogorov–Smirnov test, with Lilliefors significance correction, *p* < 0.05 in all explorations), non-parametric statistics were used. Specifically, in between-group comparisons at presentation, the Chi-square (Χ^2^) or Fisher exact test for categorical variables and the Mann-Whitney U test for continuous measures were performed. Effect sizes were also examined following the commonly accepted benchmarks. For categorical variables [degree of freedom (df) = 1], a Cramer V value greater than 0.10 indicates a small effect size, greater than 0.30 indicates a medium effect size, and ≥ 0.50 indicates a large effect size [45]. For continuous parameters, a rank biserial correlation coefficient greater than 0.10 indicates a small effect size, greater than 0.30 indicates a medium effect size, and ≥ 0.50 indicates a large effect size [46].

A mixed-design ANOVA was also carried out to investigate between-group comparisons on longitudinal psychopathological/functional outcomes measured with the PANSS, SOFAS, and HoNOS, as well as specialized PARMS treatment components. Specifically, as suggested by Blanca and coauthors [47], ANOVA remains a valid option when there are mild deviations from normality of parameters that are likely to represent real data (i.e., kurtosis and skewness indices = ± 1) [48]. Indeed, small deviations generally have a limited impact on the results, especially with balanced sample sizes. In this regard, it has been reported that the false positive rate remains substantially stable in the presence of moderate non-normality [49]. Furthermore, ANOVA was conducted in complete cases only. Indeed, except for participants who dropped out before completing the 2-year intervention program, there were no other missing data. Finally, we used the following commonly accepted benchmarks for partial eta squared (η^2^) and effect size interpretation: η^2^ > 0.01: small effect size, η^2^ > 0.06: medium effect size, and η^2^ ≥ 0.14: large effect size [50].

As for time-to-event outcome parameters (e.g., service disengagement, new suicide attempt), after having previously checked that the proportionality-of-hazards assumption was met using Schoenfeld residuals test (i.e., *p*>0.050) [51], univariate models were fitted for each outcome variable across the 2 years of follow-up, using Cox regression analysis [52]. For not time-to-event dependent parameters (e.g., current suicidal ideation, functional recovery), binary logistic regression analyses with sex as independent variable were performed [53].

## Results

**Fig. 1:**
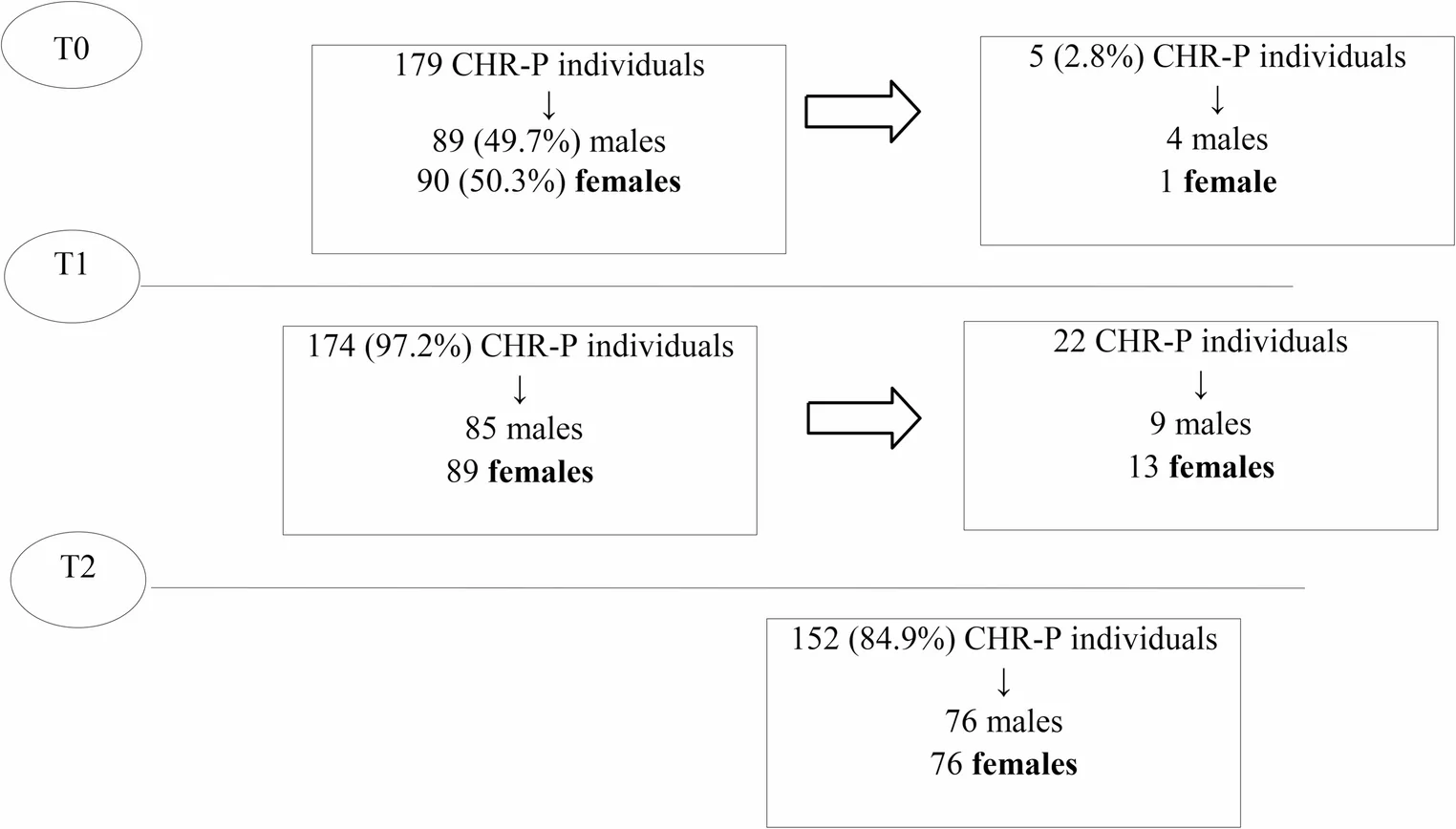
Flowchart of the CHR-P total sample and the two sex-related subgroups across the 2-year follow up period. Note. CHR-P = Clinical High Risk for Psychosis; T0 = baseline assessment; T1 = 1-year assessment time; T2 = 2-year assessment time; PARMS = Parma At-Risk Mental States. There was no intervention randomization. All CHR-P individuals enrolled in the PARMS program were considered in this research and constituted the intent-to-treat-sample for statistical analyses

Over the course of this study, 179 CHR-P patients were recruited. Of them, 89 were males (49.7%) and 90 females (50.3%). Only 5 participants dropped out of the protocol during the first year of treatment and other 22 during the second year. At the end of the 2- year follow-up period, 152 CHR-P patients completed the follow-up (84.9%). Of them, 76 were males and 76 females (Fig. 1).

### Baseline data

Between-group comparisons on sociodemographic and clinical data at baseline showed that male participants were older than women (rank biserial correlation [r]=0.203; small effect size), had a higher incidence of NEET status and a lower prevalence of being students (Cramer’s V=0.290 [df = 3]; medium effect size) (Table 1). They also showed higher prevalence rates of substance abuse (V=0.194 [df = 1]; small effect size) and borderline personality disorder (V=0.289 [df = 8]; small effect size), while women had a higher prevalence rate of eating disorder (V=0.288 [df = 8]; small effect size). Moreover, there was evidence of a sex distinction among CAARMS subgroups, with females showing a higher rate of APS (V=0.206 [df = 1]; small effect size) and males showing a higher prevalence of BLIPS (V=0.200 [df = 1]; small effect size).

**Table 1:** Baseline sociodemographic and clinical comparisons in the two CHR-P subgroups (*n* = 179)

Variable	Males (*n*=89)	Female (*n*=90)	X^2^/z	*p*
Age (at entry)	20.19±3.73	18.86±3.78	−2.353	**0.019**
Education (in years)	11.08±2.52	11.60±2.34	1.069	0.285
*Civil status*			0.139	0.999
Single	87 (97.7%)	87 (96.7%)	[0.4]	
Married/cohabitant	2 (2.3%)	3 (3.3%)	[−0.4]	
*Living status*			0.286	0.867
Alone	2 (2.2%)	3 (3.3%)	[−0.4]	
Living with partner	2 (2.2%)	2 (2.3%)	[−0.4]	
Living with parents	85 (95.6%)	85 (94.4%)	[0.5]	
*Ethnic group*			2.662	0.616
White	81 (91.0%)	80 (89.0%)	[0.4]	
Black	2 (2.3%)	4 (4.4%)	[−0.8]	
Asian	0 (0.0%)	1 (1.1%)	[−0.9]	
North African	6 (6.7%)	4 (4.4%)	[0.7]	
Hispanic	0 (0.0%)	1 (1.1%)	[−0.9]	
Migrant Status	14 (15.7%)	14 (15.6%)	0.001	0.974
*Occupation*			10.315	**0.006** ^*^
NEET	36 (40.4%)	19 (21.1%)	[2.3]	
Student	43 (48.3%)	68 (75.6%)	[−3.1]	
Employed	10 (11.3%)	3 (3.3)	[1.8]	
*Source of referral*			8.912	0.113
Primary care	44 (49.4%)	33 (36.7%)	[1.7]	
Family members	3 (3.4%)	12 (13.3%)	[−2.4]	
Self-referral	8 (9.0%)	8 (8.9%)	[0.0]	
Emergency room	17 (19.1%)	24 (26.7%)	[−1.2]	
School/Social services	8 (9.0%)	5 (5.6%)	[0.9]	
Other mental healthcare services	9 (10.1%)	8 (8.9%)	[0.3]	
First-degree family members with psychosis	32 (36.0%)	26 (28.9%)	1.020	0.312
Past hospitalization	15 (16.9%)	12 (13.3%)	0.433	0.511
Past suicide attempt	9 (10.1%)	10 (11.1%)	0.047	0.828
Previous specialist contact	45 (50.6%)	38 (42.2%)	1.251	0.263
DUI (in weeks)	45.60±48.61	47.53±49.37	0.315	0.753
Substance misuse (at entry)	22 (24.7%)	9 (10.0%)	6.771	**0.009**
*CAARMS subgroup*				
APS	62 (69.7%)	78 (86.7%)	7.592	**0.006**
BLIPS	21 (23.6%)	8 (8.9%)	7.129	**0.008**
Vulnerability	6 (6.7%)	4 (4.4%)	0.087	0.741
*DSM-5 Diagnosis*			14.995	**0.036** ^*^
Depressive disorder	25 (28.1%)	37 (41.1%)	[−1.8]	
Psychotic disorder NOS	16 (18.0%)	12 (13.3%)	[0.8]	
Schizotypal personality disorder	10 (11.2%)	16 (17.8%)	[−1.2]	
Brief psychotic disorder	16 (18.0%)	8 (8.9%)	[1.8]	
Anxiety disorder	8 (9.0%)	6 (6.7%)	[0.6]	
Borderline personality disorder	10 (11.2%)	3 (3.3%)	[2.1]	
Obsessive-compulsive disorder	4 (4.5%)	4 (4.4%)	[0.1]	
Eating disorder	0 (0.0%)	4 (4.4%)	[2.1]	
*Baseline PANSS scores*				
Positive symptoms	11.39±4.45	10.26±3.08	-0.980	0.327
Negative symptoms	21.34±8.52	17.74±6.55	−2.523	**0.012**
Disorganization	16.20±5.84	15.23±4.92	−0.787	0.431
Affect	14.76±4.85	15.88±5.43	1.132	0.257
Resistance/Excitement-activity	7.57±3.30	7.01±3.15	−1.149	0.251
Total score	74.98±19.05	68.10±15.66	2.021	**0.048**
G12 “Lack of judgment/insight”^**^	2.79±1.65	1.97±1.11	−2.149	**0.046**
Baseline SOFAS score	48.29±9.02	49.26±8.20	0.291	0.771
*Baseline HoNOS scores*				
Behavioral problems	2.91±2.19	2.44±1.97	−1.382	0.167
Impairment	2.28±2.06	2.06±1.73	−0.359	0.720
Psychiatric symptoms	8.49±3.48	8.96±2.98	1.123	0.261
Social problems	6.57±4.15	5.77±3.24	−1.095	0.273
Total score	20.26±8.74	19.22±6.99	−0.361	0.718
*Treatments*				
Baseline AP prescription	44 (49.4%)	47 (52.2%)	0.139	0.710
Equivalent dose of risperidone (mg/day)	2.78±2.20	2.59±1.64	−0.276	0.783
Baseline AD prescription	21 (23.6%)	21 (23.3%)	0.002	0.967
Baseline MS prescription	7 (7.9%)	9 (10.0%)	0.251	0.617
Baseline BDZ prescription	22 (24.7%)	19 (21.1%)	0.330	0.566
Baseline individual psychotherapy proposal	45 (50.6%)	52 (57.8%)	0.939	0.333
Baseline family psychoeducation proposal	28 (31.5%)	34 (37.8%)	0.789	0.374
Baseline case management proposal	50 (56.2%)	49 (54.4%)	0.055	0.815

Note. CHR-P = Clinical High Risk for Psychosis; NEET = Not [engaged] in Education, Employment or Training; DUI = Duration of Untreated Illness; CAARMS = Comprehensive Assessment of At-Risk Mental States; APS = Attenuated Psychotic Symptoms; BLIPS = Brief Limited Intermittent Psychotic Symptoms; DSM-5 = Diagnostic and Statistical Manual od mental disorders, 5th Edition; NOS = Not Otherwise Specified; PANSS = Positive And Negative Syndrome Scale; SOFAS = Social and Occupational Functioning Assessment Scale; HoNOS = Health of the Nation Outcome Scale; AP = Antipsychotic medication; AD = Antidepressant medication; MS = Mood Stabilizer; BDZ = Benzodiazepine. Frequencies (and percentages), mean ± standard deviation, Chi-square (X^2^) and Mann-Whitney U (z) test values are reported. Adjusted residual values are in square brackets. Statistically significant p values are in bold. ^*^Bonferroni’s corrected p values are reported (when applicable). ^**^The PANSS “Lack of judgment/insight” item is emphasized because it is not part of the five-factor model proposed by Shafer and Dazzi (2019)

Sex differences were also evident in psychopathology, with a higher PANSS “Negative Symptoms” factor score in men compared to women and a higher PANSS total score (*r*<0.300; small effect sizes). As for specific psychopathological characteristics at entry, males showed higher subscores in PANSS “Lack of judgment/Insight” item (*r*=0.191; small effect size), whereas there were no significant differences between males and females for PANSS, SOFAS and HoNOS scores, or baseline treatments provided (Table 1).

### Longitudinal data

Our univariate Cox proportional-hazard model results for 2-year time-to-event outcome parameters showed no statistically significant difference between the two CHR-P subgroups (Table 2).

**Table 2:** Univariate Cox proportional-hazard models for 2-year time-to-event outcome parameters in the two CHR-P subgroups

Variable	Males (*n* = 89)	Females (*n* = 90)	Statistic test			
			HR	95% CI		*p*
				Lower Higher		
2-year service disengagement 2-year new hospitalization 2-year new suicide attempt 2-year new self-harm behavior 2-year psychosis transition	13 (14.6%) 12 (13.5%) 5 (5.6%) 9 (10.1%) 12 (13.5%)	14 (15.5%) 13 (14.4%) 8 (8.9%) 11 (12.2%) 17 (18.9%)	1.034 1.037 1.543 1.171 1.393	0.486 0.473 0.505 0.485 0.665	2.200 2.273 4.717 2.826 2.918	0.930 0.927 0.447 0.725 0.379

Note. CHR-P = Clinical High Risk for Psychosis; HR = Hazard Ratio; 95% CI = 95% confidence intervals for HR; p = statistical significance. Significant statistical p values are in bold. All CHR-P participants are included in the analysis

Service disengagement = complete lack of contact or untraceable for at least 3 months despite a need of treatment, counted from the date of the last face-to-face meeting with the clinical staff; Suicide attempt = potentially injurious, self-inflicted behavior without a fatal outcome for which there was (implicit or explicit) evidence of intent to die, derived from direct information reported by the patient (or by a relative well informed about the facts) or documented in the clinical notes; Self-harm behavior = acts of deliberate self-harm or intoxication with alcohol or drugs, but where there was no clear intention to die.

Our binary logistic regression analysis results for 2-year not-time-to-event outcome variables between male and female subgroups didn’t show any significant differences, except for the evidence that female participants had a significant higher 2-year incidence rate of HoNOS functional remission compared to males (Table 3).

**Table 3:** Binary logistic regression analysis results for 2-year not time-to-event outcome parameters in the two CHR-P subgroups

Dependent variable	Males (*n* = 85)	Females (*n* = 89)	Statistic test				
			B (SE)	HR	95% CI		*p*
					Lower Higher		
1-year current suicidal ideation 1-year functional recovery 1-year SOFAS functional remission 1-year HoNOS functional remission 1-year PANSS symptomatic remission 1-year persistent negative symptoms 1-year new hospitalization 1-year new suicide attempt 1-year new self-harm behavior 1-year CHR-P criteria persistence 1-year psychosis transition	26 (30.6%) 12 (14.1%) 48 (56.5%) 58 (68.2%) 47 (55.3%) 11 (12.9%) 12 (14.1%) 5 (5.9%) 12 (14.1%) 39 (45.9%) 10 (11.8%)	38 (42.7%) 11 (12.4%) 57 (64.0%) 69 (77.5%) 49 (55.1%) 9 (10.1%) 11 (12.4%) 3 (3.4%) 16 (18.0%) 38 (42.7%) 13 (14.6%)	− 0.525 (0.318) 0.153 (0.448) − 0.317 (0.311) − 0.474 (0.345) 0.010 (0.305) 0.279 (0.478) 0.153 (0.448) 0.583 (0.747) − 0.288 (0.416) 0.129 (0.305) − 0.249 (0.451)	0.591 1.166 0.728 0.623 1.010 1.321 1.166 1.792 0.750 1.138 0.779	0.317 0.484 0.396 0.317 0.555 0.518 0.484 0.415 0.332 0.625 0.322	1.104 2.805 1.340 1.223 1.836 3.369 2.805 7.741 1.696 2.071 1.887	0.099 0.732 0.308 0.169 0.975 0.560 0.732 0.435 0.489 0.672 0.581
Dependent variable	Males (*n* = 76)	Females (*n* = 76)	Statistic test				
			B (SE)	HR	95% CI		p
					Lower Higher		
2-year current suicidal ideation 2-year functional recovery 2-year SOFAS functional remission 2-year HoNOS functional remission 2-year PANSS symptomatic remission 2-year persistent negative symptoms 2-year CHR-P criteria persistence	27 (35.5%) 49 (64.5%) 45 (59.2%) 58 (76.3%) 58 (76.3%) 12 (15.8%) 25 (32.9%)	32 (42.1%) 52 (68.4%) 53 (69.7%) 70 (92.1%) 56 (73.7%) 6 (7.9%) 24 (31.6%)	− 0.278 (0.334) − 0.177 (0.344) − 0.462 (0.342) −1.287 (0.504) 0.140 (0.375) 0.783 (0.529) 0.060 (0.347)	0.758 0.838 0.630 0.276 1.151 2.187 1.062	0.394 0.427 0.322 0.103 0.552 0.775 0.538	1.457 1.644 1.231 0.741 2.400 6.170 2.097	0.406 0.606 0.176 **0.011** 0.708 0.139 0.862

Note. CHR-P = Clinical High Risk for Psychosis; SOFAS = Social and Occupational FunctioningAssessment Scale; HoNOS = Health of the Nation Outcome Scale; PANSS = Positive And Negative Syndrome Scale; B = regression coefficient, SE = Standard Error; HR = Hazard Ratio; 95% CI = 95% confidence intervals for HR; p = statistical significance. Significant statistical p values are bold. Bonferroni’s corrected p values are reported. Cumulative incidence rates are reported. Only CHR-P participants who concluded the 2-year follow-up period are included in the analysis

Current suicidal ideation = BPRS item 4 score ≥ 2; Functional recovery = return to work/school; SOFAS functional remission = SOFAS score ≥ 60; HoNOS functional remission = HoNOS item 9, 10 and 11 subscores < 2; PANSS symptomatic remission = PANSS item P1, P2, P3, N1, N4, N6, G5, G9 subscores ≤ 3; Persistent negative symptoms = (a) presence of at least moderate (i.e., a score of 4 on the PANSS) for at least 3 negative symptoms or at least moderately severe (i.e., a score of 5 on the PANSS) for at least 2 negative symptoms + (b) persistence of negative symptoms for at least 6 months and for an extended period of time prior to the study beginning (e.g., at least 4 weeks) + (c) absence of relevant levels of positive symptoms, depression and extrapyramidal symptoms; Suicide attempt = potentially injurious, self-inflicted behavior without a fatal outcome for which there was (implicit or explicit) evidence of intent to die, derived from direct information reported by the patient (or by a relative well informed about the facts) or documented in the clinical notes; Self-harm behavior = acts of deliberate self-harm or intoxication with alcohol or drugs, but where there was no clear intention to die

Our mixed-design ANOVA results for clinical outcome parameters showed a longitudinal improvement in PANSS “Positive Symptoms”, “Negative Symptoms”, “Disorganization” and “Affect” dimensions, in HoNOS “Behavioral problems, “Impairment”, “Psychiatric symptoms” and “Social problems” domains, and in PANSS, HoNOS and SOFAS total scores for both subgroups, with a statistically significant “time effect” (Table 4). Moreover, there was evidence of a significant “group effect” for PANSS “Negative Symptoms” “Lack of judgment/insight” factor scores, HoNOS “Social Problems” domain scores, and SOFAS score (see also the Supplementary Materials [Figures S1 and S2] for details on profile plots and Q-Q plots of residuals). Specifically, females showed statistically relevant lower PANSS and HoNOS scores, and higher SOFAS scores over time compared to males. The same group effects were also found during the 1-year follow-up period (see the Supplementary Materials [Table S3] for details). All these statistically significant mixed-design ANOVA results had partial eta squared (η^2^) values between.041 and.066, suggesting small effect sizes.

**Table 4:** Mixed-design ANOVA results: clinical outcome parameters across the 2-year follow-up period in the two CHR-P subgroups

Variable	Time effects					Group effects				Interaction effects (time x group)			
	df	F	*p*	η2		df	F	*p*	η2	df	F	*p*	η2
*PANSS scores* Positive symptoms Negative symptoms Disorganization Affect Resistance/Excitement-activity Total score G12 Lack of judgment/insight item^*^ SOFAS score *HoNOS scores* Behavioral problems Impairment Psychiatric symptoms Social problems Total score	1.2 1.5 1.4 1.3 1.4 1.4 1.6 1.4 1.5 1.8 1.4 1.7 1.5	7.987 59.821 14.354 61.287 0.220 44.713 2.747 137.258 67.644 46.882 152.234 94.014 179.865	**0.003** ^*^ **0.001** ^*^ **0.001** ^*^ **0.001** ^*^ 0.726 **0.001** ^*^ 0.080 **0.001** ^*^ **0.001** ^*^ **0.001** ^*^ **0.001** ^*^ **0.001** ^*^ **0.001** ^*^	0.065 0.333 0.106 0.336 0.002 0.271 0.027 0.562 0.292 0.222 0.481 0.364 0.523		1 1 1 1 1 1 1 1 1 1 1 1 1	0.693 6.223 0.081 1.938 2.252 1.367 6.994 3.376 0.325 2.224 0.293 3.898 1.162	0.407 **0.014** ^*^ 0.776 0.166 0.136 0.245 **0.010** ^*^ **0.049** ^*^ 0.570 0.138 0.589 **0.047** ^*^ 0.283	0.006 0.049 0.001 0.016 0.018 0.011 0.066 0.041 0.002 0.013 0.002 0.056 0.007	1.2 1.5 1.4 1.3 1.4 1.4 1.6 1.4 1.5 1.8 1.4 1.7 1.5	1.296 0.851 1.457 0.131 0.551 0.825 0.060 0.456 0.308 1.385 1.379 0.466 0.639	0.264 0.400 0.236 0.782 0.577 0.402 0.906 0.567 0.678 0.252 0.251 0.602 0.485	0.011 0.007 0.012 0.001 0.005 0.007 0.001 0.004 0.002 0.008 0.008 0.003 0.004
Variable (Group effects)		EMM (SE)											
		Males (*n* = 76)			Females (*n* = 76)								
T0 PANSS Negative symptoms T1 PANSS Negative symptoms T2 PANSS Negative symptoms		21.340 (1.026) 16.415 (0.999) 15.377 (0.853)			17.739 (0.899) 13.957 (0.875) 12.986 (0.747)								
T0 G12 PANSS “Lack of Judgment/Insight T1 G12 PANSS “Lack of Judgment/Insight T2 G12 PANSS “Lack of Judgment/Insight		2.622 (0.201) 2.444 (0.200) 2.356 (0.200)			1.946 (0.180) 1.804 (0.179) 1.750 (0.180)								
T0 SOFAS score T1 SOFAS score T2 SOFAS score		46.240 (1.170) 57.760 (1.866) 60.880 (1.897)			49.525 (1.078) 62.475 (1.718) 63.932 (1.746)								
T0 HoNOS Social problems T1 HoNOS Social problems T2 HoNOS Social problems		6.568 (0.407) 4.815 (0.340) 4.160 (0.311)			5.776 (0.398) 3.718 (0.331) 3.376 (0.311)								

Note. ANOVA = analysis of variance; CHR-P = Clinical High Risk for Psychosis; HoNOS = Health of the Nation Outcome Scale; PANSS = Positive And Negative Syndrome Scale; SOFAS = Social and Occupational Functioning Assessment Scale; df = degrees of freedom; F = F statistic value; p = statistical significance; η2 = partial eta squared; EMM = Estimated Marginal Mean; SE = Standard Error. As all Mauchly’s tests of sphericity are statistically significant (*p* < 0.05), Greenhouse–Geisser corrected degrees of freedom to assess the significance of the corresponding F value are used. Statistically significant p values are in bold. ^*^Bonferroni’s corrected p values are reported. Only CHR-P participants who concluded the 2-year follow-up period are included in the analysis. ^*^The PANSS “Lack of judgment/insight” item is emphasized because it is not part of the five-factor model proposed by Shafer and Dazzi (2019)

As for specialized treatments provided in the PARMS program across the 2-years follow-up period, our mixed-design ANOVA showed a “time effect” for all the psychosocial interventions, with a longitudinal increase in the number of sessions provided during time. Conversely, there was no significant result related to psychopharmacological therapy over time (Table 5). However, no group or interaction (time x group) effects were found in terms of PARMS treatments received longitudinally.

**Table 5:** Mixed-design ANOVA results: specialized treatment components of the PARMS program across the 2-year follow-up period in the two CHR-P subgroups

Variables	Time effects				Group effects				Interaction effects (time x group)			
	df	F	*p*	η2	df	F	*p*	η2	df	F	*p*	η2
Equivalent dose of risperidone (mg/day) Equivalent dose of fluoxetine (mg/day) Equivalent dose of lorazepam (mg/day) Individual psychotherapy sessions Family psychoeducation sessions Case management sessions	1.9 1.7 1.4 1 1 1	0.496 0.552 0.405 108.861 73.082 58.746	0.609 0.546 0.598 **0.001** ^*^ **0.001** ^*^ **0.001** ^*^	0.003 0.004 0.003 0.422 0.328 0.281	1 1 1 1 1 1	2.110 2.076 1.339 1.613 0.476 1.089	0.148 0.152 0.249 0.206 0.491 0.298	0.014 0.014 0.009 0.011 0.003 0.007	1.9 1.7 1.4 1 1 1	0.490 0.491 0.518 1.917 0.023 1.635	0.613 0.579 0.536 0.168 0.879 0.203	0.003 0.003 0.003 0.013 0.001 0.011

Note. ANOVA = analysis of variance; PARMS = Parma At-Risk Mental States; CHR-P = Clinical High Risk for Psychosis; df = degrees of freedom; F = F statistic value; p = statistical significance; η2 = partial eta squared. As all Mauchly’s tests of sphericity are statistically significant (*p* < 0.05), Greenhouse–Geisser corrected degrees of freedom to assess the significance of the corresponding F value are used. Statistically significant p values are in bold. ^*^Bonferroni’s corrected p values are reported. Only CHR-P participants who concluded the 2-year follow-up period were included in the analysis

## Discussion

This study aimed to investigate sex differences in a sample of males and females at CHR-P treated within an Italian EIP service, in terms of socio-demographic and psychopathological features at baseline, and in clinical and functional outcomes across a two-year follow-up period. There were no specific a-priori hypotheses, we only investigated the presence of potential sex differences at baseline and as for longitudinal outcomes to evaluate new data confirming or contradicting the scarce scientific evidence on this topic published so far in the current literature.

The *prevalence* of males and females at entry was similar. This represents a difference with most samples of individuals at CHR-P already examined, where there was a higher prevalence of males, especially migrants [2, 54, 55]. This discrepancy may be related to geographic specificities and differences in the characteristics of EIP programs. In this regard, many comparable samples also included adolescents and young adults with FEP.

In line with previous findings [6, 7, 56], our results showed that males had a lower occupation rate, despite being older in age. Indeed, they showed a higher incidence of NEET status and a lower prevalence of being students. This suggests the presence of more disadvantaged *functional conditions* for men already at presentation in CHR-P services. Furthermore, there was a sex difference in terms of *clinical presentation*, with a higher percentage of females showing APS and a higher percentage of males showing BLIPS and substance abuse at entry. These findings support different between-sex clinical severity levels at presentation, with men manifesting more overt psychotic symptoms and more problematic behaviors, particularly substance abuse [8]. This is further confirmed by results on psychopathological features. Indeed, males showed higher severity levels in PANSS negative symptoms, PANSS total score, and PANSS “Lack of judgment/insight” compared to women. Overall considered, these results are also in line with those reported in men with FEP, suggesting the presence of more serious clinical presentations with lower premorbid functioning and less awareness of illness in males compared to females [2, 7, 10]. Therefore, male sex can be considered a potential unfavorable indicator of critical psychopathological and functional conditions at the time of presentation in EIP services, not only in full-blown FEP but already in CHR-P mental states. In interpreting and comparing our results, we should also consider the findings of a recent and interesting study suggesting the importance of recruitment sources for CHR-P sample characteristics [57]. According to the authors, variations in the male/female ratio in CHR-P studies can have a significant impact on research findings. Specifically, clinical heterogeneity may be partly related to differences in age, sex, and recruitment source. Indeed, their statistical analyses revealed a significant shift towards older age and a female predominance among participants recruited through internet self-referrals.

Our *longitudinal results* overall showed no significant inter-group difference both for 2-year time-to-event and for 2-year not-time-to-event outcome variables. Moreover, the two CHR-P subgroups also had similar psychosis conversion rates overtime. Previous findings on this matter are quite conflicting, with some authors observing analogue rates while others reporting higher psychosis transition incidence in men [6, 9, 10].

In the current study, the only relevant intergroup difference was in terms of 2-year HoNOS functional remission, which were higher in females compared to males. This supports a better functional remission overtime in CHR-P women, in line with what was reported in women with FEP [58]. This longitudinal finding was not confirmed int terms of SOFAS functional remission. However, HoNOS scores assessed a slightly broader and more specific range of socio-occupational information including not only problems related to interpersonal relationships and employment, but also activity of daily living and housing/living conditions. Furthermore, the fact that women had a lower rate of NEET status at entry could partly confound this association. Therefore, further studies are needed to better evaluate functional remission using more appropriate assessment tools, including self-reported ones.

Our mixed-design ANOVA results overall support the time effects of PARMS specialized early interventions in both males and females across the 2-year follow-up period [59]. The evidence of a “group effect” regarding PANSS “Negative Symptoms” and “Lack of Judgment/Insight” scores, as well as regarding HoNOS “Social Problems” domain subscores and SOFAS scores, suggests a higher general severity in psychopathology and functioning in the male subgroup compared to the female one across the entire follow-up period, despite the evidence of a clinical improvement in both subgroups. This substantially confirms what was previously reported in men with FEP [1, 4, 6, 7, 56]. In this regard, specific treatments or strategies (such as increasing the intensity of psychosocial interventions or extending the duration of EIP treatment beyond two years) could longitudinally improve functional outcomes in both subgroups, including male participants.

A significant time effect was also found in all psychosocial interventions, further supporting the PARMS program’s clinical usefulness in both subgroups, independently from sex. Indeed, the increase of individual psychotherapy, family psychoeducation and case management sessions is in line with the longitudinal clinical improvement observed in the total sample overtime. Moreover, the absence of group effects or interaction effects further supports the evidence of similar time effects of our interventions between the two subgroups. No sex difference was also found in terms of intensity of psychosocial interventions provided, as well as in terms of doses and prescriptions of psychotropic medications. In this regard, although meta-analytic findings suggest that sex differences do not play a crucial role in any type of psychotherapy [60], sex issues might play an indirect role. Interestingly, a pilot study conducted on 40 patients with mental disorder [61] indicated that although males and females equally valued the importance of therapeutic alliance between the therapist and client, men tended to be more sensitive to factors that inhibit the therapeutic process than women. Particularly, men more frequently than women experienced a sense of “something missing” in therapy sessions, such as difficulties obtaining validation from the therapist, unsuccessful attempt to find a solution, or an inability to adopt healthy coping mechanisms. These feelings of missing elements, which had a greater impact on men’s satisfaction, may vary from individual to individual, due to the subjective nature of the psychotherapy experience and everyone’s idea of a successful therapeutic experience. However, to our knowledge, no study examining sex differences in psychotherapy for CHR-P subjects has been reported in the literature to date. Therefore, future research on this topic is welcome.

However, the interpretation of better longitudinal functional outcomes in women should be contextualized by also considering potential age-related confounding factors. Indeed, our female participants were younger at baseline, and previous literature suggested that earlier intervention and shorter illness duration may be associated with better outcomes [62, 63]. In this sense, it remains uncertain whether the observed differences are attributable to sex per se or may partly reflect earlier clinical presentation and greater engagement in treatment in women. Similarly, although no intergroup difference was found in SOFAS scores at presentation, CHR-P males showed a higher baseline prevalence rate of NEET, which may indicate a decline in functioning early in the trajectory. Therefore, observed differences in functional remission may also partly reflect pre-existing baseline disparities rather than differentiated longitudinal trajectories. In this sense, we must be cautious in emphasizing baseline sex differences and examining true sex-time trajectory effects, despite the largely non-significant longitudinal interaction effects observed across outcomes. However, the reported intersex difference in terms of 2-year HoNOS functional remission remained statistically significant even in the multivariate analysis that considered age and NEET as potential confounding parameters (see Supplementary Materials [Table S4] for details). Finally, to be fair, overall considered, our statistically significant results showed small effect sizes and should be interpreted with caution. Further studies with larger CHR-P samples are needed to replicate our findings and draw more robust conclusions.

### Limitations

Although this investigation had some important strengths (such as examining sex differences in a sample of individuals with CHR-P with the aim of identifying possible different risk trajectories), some limitations must also be considered. First, the sample size was quite small, although only a minimal percentage of patients dropped out over the follow-up. Specifically, this is a relevant issue, given the focus on sex-stratified longitudinal and time-to-event analyses, where attrition and subgrouping may substantially reduce statistical power. Because our original protocol did not define recruitment targets and retention assumptions, current analyses should be considered exploratory. Indeed, the largely null findings observed in several longitudinal and transition-related outcomes may reflect insufficient statistical power rather than true equivalence between sexes. Furthermore, the number of transitions and time-to-event outcomes are relatively limited, which may reduce the stability and precision of Cox regression estimates. Therefore, future follow-up studies in larger and multicenter CHR-P samples are needed. However, because selective service disengagement and drop-out may bias longitudinal findings, we examined whether attrition was associated with sex or other baseline clinical/functional characteristics (for details see Supplementary Materials [Table S5]) and found no intergroup differences.

Second, this study focused mainly on sociodemographic and psychopathological features, without investigating any cognitive aspect, as reported in other studies. Furthermore, the age range (12–25 years) encompassed considerable developmental heterogeneity, including adolescence and early adulthood, and developmental stage was not specifically examined in the analyses. However, it should be considered that adolescent mental health services are focusing on treating young adults up to the age of 25, taking into account evidence showing that the brain continues to develop up to that age, and also considering that many of the issues affecting young adults are also relevant and current for older adolescents [64].

Third, no comparison was made between sex differences in the CHR-P sample and those in comparable samples of healthy individuals. This could be crucial to undermine the actual effectiveness of CHR-P treatments considering sex [65]. Rather, we provided a descriptive account of the program’s outcomes, which requires future randomized clinical trials with control groups. In this regard, the naturalistic observational design of this research and the absence of a comparison group preclude the possibility of drawing causal conclusions regarding treatment efficacy. Indeed, the improvement observed over time may reflect several alternative explanations, including natural fluctuation of symptoms, regression towards the mean, spontaneous remission, repeated measures effects, or selective retention effects [66].

Fourth, the follow-up period we considered for this study was of 2 years, while a longer follow-up period would be necessary to evaluate the long-term effects of the carried-out interventions, as well as to better investigate any difference in psychopathological trajectories between males and females. Fifth, no gender diversity was explored. Therefore, if some of these differences are environmentally mediated, the same assumptions about how environmental factors act on latent risk might not hold for youth who experience themselves and present to their communities as neither male nor female. Further research on this topic is needed.

Sixth, the use of mixed-design ANOVA may raise concerns regarding the handling of missing repeated-measures data, especially because analyses were conducted on complete cases only. In this respect, some current longitudinal psychiatric analyses frequently rely on linear mixed-effects models rather than repeated-measures ANOVA because they better accommodate incomplete repeated-measure datasets and non-random missingness. However, because there were no other missing data except for CHR-P participants who dropped out before completing our 2-year intervention package, the choice to use mixed-design ANOVA was related to a relative balanced study design and dataset across follow-ups and the “rule of simplicity” in statistics, which suggests selecting the simplest model if multiple models fit the data adequately and data dependence is negligible [67].

Seventh, both the Cox and logistic regression analyses were univariate, with sex entered as the sole predictor. Therefore, the absence of adjustment for potential confounding variables (e.g., age, baseline symptom severity, daily functioning) may substantially limit interpretation of observed sex differences. This issue is particularly important because baseline differences between males and females were already present at presentation. Taking this into account, a cautious interpretation of our results is necessary. However, the reported intersex difference in terms of 2-year HoNOS functional remission, which were greater in females than in males, remained statistically significant even in the multivariate analysis adjusting for those potential confounding parameters that were different between the two subgroups at entry (see Supplementary Materials [Table S4] for details).

Eight, other potential selection and generalizability biases could be related to recruitment within a single Italian service. Indeed, sociodemographic composition, referral pathways, health system organization, and local service characteristics can substantially influence both sex distribution and longitudinal outcomes, thereby limiting external validity and comparability with other CHR-P cohorts. Finally, we did not examine the potential need of developing differentiated pharmacological/psychosocial interventions based on sex. This should be evaluated in future research.

## Conclusions

The results of this study showed the presence of crucial differences in clinical presentation between CHR-P men and women, with more severe psychopathology and lower functioning at presentation in males, although not as marked as the ones reported in FEP patients [5]. However, these disparities do not appear to significantly influence the risk of transition to psychosis or the clinical/functional outcomes when specialized early intervention treatments are provided. This supports the contribution of the PARMS program in terms of effects over time on clinical and functional outcomes in both males and females, with no significant prognostic differences, except for a better functional remission showed by women over the follow-up. The result of a better functional remission for females extends throughout our follow-up and confirms the clinical utility of specialized interventions in both sexes for the entire duration of the two-year intervention period, also in the context of Italian public mental healthcare services. Further research should explore better the potential implications of sex on psychosis trajectory, starting from the Clinical High-Risk psychopathology, and how these could support the development of sex-specific interventions.

## Supplementary Information

Below is the link to the electronic supplementary material.

Supplementary Material 1 (DOCX 300 KB)

## Data Availability

The data that support the findings of this research are available on reasonable request from the corresponding author. The data are not publicly available due to privacy and/or ethical restrictions.

## References

[1] Ochoa S, Usall J, Cobo J, Labad X, Kulkarni J (2012) Gender differences in schizophrenia and first-episode psychosis: a comprehensive literature review. Schizophr Res Treatment 2012:916198. 10.1155/2012/916198 PMC3420456 22966451

[2] Hui CL, Leung CM, Chang WC, Chan SK, Lee EH, Chen EY (2016) Examining gender difference in adult-onset psychosis in Hong Kong. Early Interv Psychiatry 10:324–333. 10.1111/eip.12167 25130368

[3] Carter B, Wootten J, Archie S, Terry AL, Anderson KK (2022) Sex and gender differences in symptoms of early psychosis: a systematic review and meta-analysis. Arch Womens Ment Health 25:679–691. 10.1007/s00737-022-01247-3 35748930

[4] Ventura J, Subotnik KL, Han S, Hellemann GS, Green MF, Nuechterlein KH (2023) The relationship between sex and functional outcome in first-episode schizophrenia: the role of premorbid adjustment and insight. Psychol Med 53: 6878–6887. 10.1017/S0033291723000442 PMC10600815 38314778

[5] Pelizza L, Ricci C, Leuci E, Quattrone E, Palmisano D, Pupo S, Paulillo G, Pellegrini C, Pellegrini P, Menchetti M (2025) Sex comparisons on the beneficial effects of an early intervention program in a patients’ cohort with first episode psychosis: what effectiveness in women? Arch Womens Ment Health 28: 1151–1166. 10.1007/s00737-025-01566-1 PMC12436571 39971780

[6] Barajas A, Ochoa S, Obiols JE, Lalucat-Jo L (2015) Gender differences in individuals at high-risk of psychosis: a comprehensive literature review. ScientificWorldJournal 2015:430735. 10.1155/2015/430735 PMC4312997 25685840

[7] Willhite RK, Niendam TA, Bearden CE, Zinberg J, O’Brien MP, Cannon TD (2008) Gender differences in symptoms, functioning and social support in patients at ultra-high risk for developing a psychotic disorder. Schizophr Res 104:237–245. 10.1016/j.schres.2008.05.019 PMC2612126 18573639

[8] Fusar-Poli P, Salazar de Pablo G, Correll CU, Meyer-Lindenberg A, Millan MJ, Borgwardt S, Galderisi S, Bechdolf A, Pfennig A, Kessing LV, van Amelsvoort T, Nieman DH, Domschke K, Krebs MO, Koutsouleris N, McGuire P, Do KQ, Arango C (2020) Prevention of psychosis: advances in detection, prognosis, and intervention. JAMA Psychiatry 77:755–765. 10.1001/jamapsychiatry.2019.4779 32159746

[9] He XY, Wang SB, Hou CL, Guo LL, Huang ZH, Zhao QN, Li D (2023) Meta-analysis of gender differences in transition prevalence among individuals at clinical high risk of psychosis. Asian J Psychiatr 86:103639. 10.1016/j.ajp.2023.103639 37307702

[10] Chan KN, Chang WC, Ng CM, Lee HC, Chan SI, Chiu SY, Wong CF, Wo SF, Lee HM, Chan KW, Wong MC, Chan KL, Yeung WS, Chan CWH, Choy LW, Chong SY, Siu MW, Lo TL, Yan WC, Ng MK, Poon LT, Pang PF, Lam WC, Wong YC, Chung WS, Mo YM, Lui SY, Hui LM, Chen EYH (2021) Sex differences in symptom severity, cognition and psychosocial functioning among individuals with at-risk mental state for psychosis. Early Interv Psychiatry 16:61–68. 10.1111/eip.13131 33590717

[11] Menghini-Müller S, Studerus E, Ittig S, Valmaggia LR, Kempton MJ, van der Gaag M, de Haan L, Nelson B, Bressan RA, Barrantes-Vidal N, Jantac C, Nordentoft M, Ruhrmann S, Sachs G, Rutten BP, van Os J, Riecher-Rössler A, EU-GEI High Risk Study Group (2020) Sex differences in cognitive functioning of patients at-risk for psychosis and healthy controls: results from the European Gene-Environment Interactions study. Eur Psychiatry 63: e25. 10.1192/j.eurpsy.2019.10 PMC7315874 32167444

[12] Bucci P, Giordano GM, Mucci A, Rocca P, Rossi A, Bertolino A, Aguglia E, Altamura C, Amore M, Bellomo A, Biondi M, Carpiniello B, Cascino G, Dell’Osso L, Fagiolini A, Giuliani L, Marchesi C, Montemagni C, Pettorruso M, Pompili M, Rampino A, Roncone R, Rossi R, Siracusano A, Tenconi E, Vita A, Zappegno P, Galderisi S, Maj M, Italian Network for Research on Psychosis (2023) Sex and gender differences in clinical and functional indices in subjects with schizophrenia and healthy controls: data from the baseline and 4-year follow-up studies of the Italian Network for Research on Psychoses. Schizophr Res 251: 94–107. 10.1016/j.schres.2022.12.021 36610377

[13] Fresan A, Apiquian R, Ulloa RE, Loyzaga C, Nicolini H, Gomez L (2003) Premorbid functioning by gender and its relationship with duration of untreated psychosis in first episode psychosis. Actas Esp Psiquiatr 31: 53–58. https://europepmc.org/article/MED/12677467. 12677467

[14] Pelizza L, Leuci E, Quattrone E, Paulillo G, Pellegrini P (2023) The “Parma At-Risk mental states” (PARMS) program: general description and process analysis after 5 years of clinical activity. Early Intervention in Psychiatry 17:625–635. 10.1111/eip.13399 36639137

[15] Yung AR, Yuen HP, McGorry PD, Phillips LJ, Kelly D, Dell’Olio M, Francey SM, Cosgrave EM, Killackey E, Stanford C, Godfrey K, Buckby J (2005) Mapping the onset of psychosis: the Comprehensive Assessment of At-Risk Mental States. Aust N Z J Psychiatry 39: 964–971. 10.1080/j.1440-1614.2005.01714.x 16343296

[16] Salazar de Pablo G, Woods SW, Drymonitou G, de Diego H, Fusar-Poli P (2021) Prevalence of individuals at clinical high risk of psychosis in the general population and clinical samples: systematic review and meta-analysis. Brain Sci 11:1544. 10.3390/brainsci11111544 PMC8615691 34827543

[17] Pelizza L, Azzali S, Paterlini F, Garlassi S, Scazza I, Chiri LR, Poletti M, Pupo S, Raballo A (2021) Anhedonia in the psychosis risk syndrome: state and trait characteristics. Psychiatr Danub 33: 36–47. 10.24869/psyd.2021.36. 33857038

[18] Pelizza L, Poletti M, Azzali S, Garlassi S, Scazza I, Paterlini F, Chiri LR, Pupo S, Raballo A (2021) Subjective experience of social cognition in young people at ultra-high risk of psychosis: a 2-year longitudinal study. Nord J Psychiatry 75:97–108. 10.1080/08039488.2020.1799430 32762506

[19] Orsini A, Pezzuti L (2013) WAIS-IV (Wechsler Adult Intelligence Scale, IV edizione), versione italiana. Giunti Psychometrics, Firenze

[20] Orsini A, Picone L, Pezzuti L (2012) WISC-IV (Wechsler Intelligence Scale for Children, IV edizione), versione italiana. Giunti Psychometrics, Firenze

[21] Wing JK, Beevor AS, Curtis RH, Park SB, Hadden S, Burns A (1998) Health of the nation outcome scales (HoNOS): research and development. Br J Psychiatry 172:11–18. 10.1192/bjp.172.1.11 9534825

[22] Kay SR, Fiszbein A, Opler LA (1987) The positive and negative syndrome scale (PANSS) for schizophrenia. Schizophr Bull 13:261–276. 10.1093/schbul/13.2.261 3616518

[23] Saraswat N, Rao K, Subbakrishna DK, Gangadhar BN (2006) The social occupational functioning scale (SOFS): a brief measure of functional status in persons with schizophrenia. Schizophr Res 81:301–309. 10.1016/j.schres.2005.09.008 16256309

[24] Raballo A, Semrov E, Bonner Y, Simmons M (2013) Traduzione e adattamento della CAARMS (Comprehensive Assessment of At-Risk Mental States). Centro Stampa della Regione Emilia-Romagna, Bologna

[25] Pelizza L, Paterlini F, Azzali S, Garlassi S, Scazza I, Pupo S, Simmons M, Nelson B, Raballo A (2019) The approved Italian version of the comprehensive assessment of at-risk mental states (CAARMS-ITA): field test and psychometric features. Early Intervention in Psychiatry 13:810–817 10.1111/eip.12669 29696795

[26] Belvederi Murri M, Ferrara M, Imbesi M, Leuci E, Marchi M, Musella V, Natali A, Neri A, Ragni S, Saponaro A, Tarricone I, Tullini A, Starace F, Early Psychosis Working Group (2023) A public early intervention approach to first-episode psychosis: treated incidence over 7 years in the Emilia-Romagna region. Early Intervention in Psychiatry 17:724–736. 10.1111/eip.13437 37221039

[27] Kortrijk HE, Mulder CL, Drukker M, Wiersma D, Duivenvoorden HJ (2012) Duration of assertive community treatment and the interpretation of routine outcome data. Aust N Z J Psychiatry 46:240–248. 10.1177/0004867411433214 22391281

[28] Lora A, Bai G, Bianchi S, Bolongaro G, Civenti G, Erlicher A, Maresca G, Monzani E, Panetta B, Von Morgen D, Rossi F, Torri V, Morosini P (2001) La versione italiana della HoNOS (Health of the Nation Outcome Scales), una scala per la valutazione della gravità e dell’esito nei servizi di salute mentale. Epidemiol Psichiatr Soc 10:198–212

[29] Leuci E, Pelizza L, Landi G, Quattrone E, Maestri D, Azzali S, Pelosi A, Ceroni P, Soncini C, Daolio MC, Paulillo G, Raballo A, Pellegrini P (2022) Personal health budget in patients with first episode psychosis: a new rehabilitation model based on a community care system in Italy. Early Interv Psychiatry 16:221–230. 10.1111/eip.13145 33754490

[30] Scazza I, Pelizza L, Azzali S, Garlassi S, Paterlini F, Chiri LR, Poletti M, Pupo S, Raballo A (2022) Aberrant salience in first-episode psychosis: Longitudinal stability and treatment-response. Early Interv Psychiatry 16:912–919. 10.1111/eip.13243 34786863

[31] Shafer A, Dazzi F (2019) Meta-analysis of the Positive and Negative Syndrome Scale (PANSS) factor structure. J Psychiatr Res 115:113–120. 10.1016/j.jpsychires.2019.05.008 31128501

[32] Andreasen NC, Carpenter WT Jr, Kane JM, Lasser RA, Marder SR, Weinberger DR (2005) Remission in schizophrenia: proposed criteria and rationale for consensus. Am J Psychiatry 162:441–449. 10.1176/appi.ajp.162.3.441 15741458

[33] Schennach-Wolff R, Jäger M, Seemüller F, Obermeier M, Messer T, Laux G, Pfeiffer H, Naber D, Schmidt LG, Gaebel W, Huff W, Heuser I, Maier W, Lemke MR, Rüther E, Buchkremer G, Gastpar M, Möller HJ, Riedel M (2009) Defining and predicting functional outcome in schizophrenia and schizophrenia spectrum disorders. Schizophr Res 113:210–217. 10.1016/j.schres.2009.05.032 19560901

[34] Silva MA, Restrepo D (2019) Functional recovery in schizophrenia. Rev Colombiana Psiquiatr 48:252–260. 10.1016/j.rcp.2017.08.004 31779876

[35] Gunnes M, Thaulow K, Kaspersen SL, Jensen C, Ose SO (2025) Young adults not in education, employment, or training (NEET): a global scoping review. BMC Public Health 25:3394. 10.1186/s12889-025-24781-y PMC12505988 41063079

[36] Schultze-Lutter F, Rahman J, Ruhrmann S, Michel C, Schimmelmann BG, Maier W, Klosterkötter J (2015) Duration of unspecific prodromal and clinical high risk states, and early help-seeking in first-admission psychosis patients. Soc Psychiatry Psychiatr Epidemiol 50: 1831–1841. 10.1007/s00127-015-1093-3 26155901

[37] Silverman MM, Berman AL, Sanddal ND, O’carroll PW, Joiner TE (2007) Rebuilding the Tower of Babel: a revised nomenclature for the study of suicide and suicidal behaviors - Part 2: suicide-related ideations, communications, and behaviors. Suicide Life Threat Behav 37:264–277. 10.1521/suli.2007.37.3.264 17579539

[38] Robson E, Greenwood K (2022) Rates and predictors of disengagement and strength of engagement for people with a first episode of psychosis using early intervention services: a systematic review of predictors and meta-analysis of disengagement rates. Schizophr Bull Open 3:sgac012. 10.1093/schizbullopen/sgac012 PMC11205872 39144778

[39] Pelizza L, Maestri D, Leuci E, Quattrone E, Azzali S, Paulillo G, Pellegrini P (2022) Individual psychotherapy can reduce suicidal ideation in first episode psychosis: further findings from the 2-year follow-up of the “Parma Early Psychosis” programme. Clin Psychol Psychother 29:982–989. 10.1002/cpp.2678 34704336

[40] First MB, Williams JBW, Karg RS, Spitzer RL (2016) SCID-5-CV: Structured Clinical Interview for DSM-5 mental disorders, Clinical Version. American Psychiatric Association Publishing, Washington (DC)

[41] Gramble NK, Pruski M (2018) Teleology and defining sex. New Bioeth 24:176–189. 10.1080/20502877.2018.1468601 29733759

[42] Poletti M, Azzali S, Paterlini F, Garlassi S, Scazza I, Chiri LR, Pupo S, Raballo A, Pelizza L (2021) Familiarity for serious mental illness in help-seeking adolescents at clinical high risk of psychosis. Front Psychiatry 11:552282. 10.3389/fpsyt.2020.552282 PMC7819871 33488412

[43] SPSS Inc (2010) Statistical Package for Social Science, version 15.0 for Windows. SPSS Press, Chicago (IL)

[44] Armstrong RA (2014) When to use the Bonferroni correction. Ophthalmic Physiol Opt 34:502–508. 10.1111/opo.12131 24697967

[45] Shekari Z, Sadeghian Afarani R, Fatorehchy S, Bakhshi E, Shahshahani S, Mousavi E (2024) Relationship between postural asymmetry, balance, and pain in children with spastic cerebral palsy. Pediatr Neurol 155:84–90. 10.1016/j.pediatrneurol.2024.03.018 38608553

[46] Schubert AL, Steinhilber M, Kang H, Quintana DS (2025) Improving statistical reporting in psychology. Commun Psychol 3:156. 10.1038/s44271-025-00356-w PMC12618885 41238797

[47] Blanca MJ, Alarcón R, Arnau J, Bono R, Bendayan R (2017) Non-normal data: is ANOVA still a valid option? Psicothema. 10.7334/psicothema2016.383 29048317

[48] Kim HY (2013) Statistical notes for clinical researchers: assessing normal distribution (2) using skewness and kurtosis. Restor Dent Endod 38:52–52. 10.5395/rde.2013.38.1.52 PMC3591587 23495371

[49] Lix LM, Keselman HJ (2004) Multivariate tests of means in independent groups designs: effects of covariance heterogeneity and nonnormality. Eval Health Prof 27:45–69. 10.1177/0163278703261213 14994559

[50] Correll J, Mellinger C, Pedersen EJ (2022) Flexible approaches for estimating partial eta squared in mixed-effects models with crossed random factors. Behav Res Methods 54:1626–1642. 10.3758/s13428-021-01687-2 34561820

[51] In J, Lee DK (2019) Survival analysis: Part II – applied clinical data analysis. Korean J Anesthesiol 72:441–457. 10.4097/kja.19183 PMC6781220 31096731

[52] Zhang Z, Reinikainen J, Adeleke KA, Pieterse ME, Groothuis-Oudshoorn CGM (2018) Time-varying covariates and coefficients in Cox regression models. Ann Transl Med 6:121. 10.21037/atm.2018.02.12 PMC6015946 29955581

[53] Harris JK (2021) Primer on binary logistic regression. Fam Med Community Health 9(Suppl. 1):e001290. 10.1136/fmch-2021-001290 PMC8710907 34952854

[54] Cotton SM, Lambert M, Schimmelmann BG, Foley DL, Morley KI, McGorry PD, Conus P (2009) Gender differences in premorbid, entry, treatment, and outcome characteristics in a treated epidemiological sample of 661 patients with first episode psychosis. Schizophr Res 114:17–24. 10.1016/j.schres.2009.07.002 19635660

[55] Køster A, Lajer M, Lindhardt A, Rosenbaum B (2008) Gender differences in first episode psychosis. Soc Psychiatry Psychiatr Epidemiol 43:940–946. 10.1007/s00127-008-0384-3 18574541

[56] Giordano GM, Bucci P, Mucci A, Pezzella P, Galderisi S (2021) Gender differences in clinical and psychosocial features among persons with schizophrenia: a mini review. Front Psychiatry 12:789179. 10.3389/fpsyt.2021.789179 PMC8727372 35002807

[57] Farina EA, Mourgues-Codern C, Stimler K, Kenney J, Saxena A, Mukhtar H, Addington J, Bearden CE, Cadenhead KS, Cannon TD, Cornblatt B, Ellman L, Gold J, Keshavan M, Mathalon DH, Mittal VA, Perkins DO, Schiffman J, Silverstein SM, Strauss GP, Stone WS, Walker EF, Waltz J, Corlett P, Powers AR, Woods SW (2025) Shift in sex and age of individuals at a clinical high risk (CHR) for psychosis: relation to differences in recruitment methods and effect on sample characteristics. Schizophr 11:123. 10.1038/s41537-025-00663-5 PMC12501016 41053030

[58] Montemagni C, Frieri T, Blandamura A, Villari V, Rocca P (2015) Gender differences in 353 inpatients with acute psychosis: the experience of one Psychiatric Emergency Service of Turin. Psychiatry Res 227:192–197. 10.1016/j.psychres.2015.03.035 25890692

[59] Pelizza L, Leuci E, Quattrone E, Azzali S, Paulillo G, Pupo S, Pellegrini P, Menchetti M (2023) Longitudinal evaluation on negative symptoms in young people at ultra-high risk (UHR) of psychosis: results from a 2-year follow-up study in a real-world care setting. Eur Arch Psychiatry Clin Neurosci 273:1761–1771. 10.1007/s00406-023-01600-2 37029806

[60] Staczan P, Schmuecker R, Koehler M, Berglar J, Crameri A, von Wyl A, Koemeda-Lutz M, Schulthess P, Tschuschke V (2017) Effects of sex and gender in ten types of psychotherapy. Psychother Res 27:74–88. 10.1080/10503307.2015.1072285 26291131

[61] Arora S, Bhatia S (2022) Gender differences in factors that facilitate successful therapeutic progress and outcome: a pilot study. Couns Psychother Res 22:1030–1040. 10.1002/capr.12564

[62] Shadowen N, Meehan Z, Webb C, Fowles T, Beveridge R (2023) Early intervention in youth psychosis: novel approaches to understanding change. Psychiatry Res 326:115269. 10.1016/j.psychres.2023.115269 37331067

[63] Poletti M, Preti A, Raballo A (2026) Duration of aactive attenuated psychosis (DAAP): extending time-based illness modelling to the clinical high-risk state. Schizophr Res 293:27–29. 10.1016/j.schres.2026.03.019 41887093

[64] Aberizk K, Addington JM, Bearden CE, Cadenhead KS, Cannon TD, Cornblatt BA, Keshavan M, Mathalon DH, Perkins DO, Stone WS, Tsuang MT, Woods SW, Walker EF, Ku BS (2024) Relations of lifetime perceived stress and basal cortisol with hippocampal volume among healthy adolescents and those at clinical high risk for psychosis: a structural equation modeling approach. Biol Psychiatry 96:401–411. 10.1016/j.biopsych.2023.11.027 PMC11166888 38092185

[65] Pelizza L, Leuci E, Quattrone E, Azzali S, Pupo S, Paulillo G, Menchetti M, Pellegrini P (2024) Adverse outcome analysis in people at clinical high risk for psychosis: results from a 2-year Italian follow-up study. Soc Psychiatry Psychiatr Epidemiol 59:1177–1191. 10.1007/s00127-023-02597-8 38091031

[66] Tang SX, Spilka MJ, John M, Birnbaum ML, Saito E, Berretta SA, Behbehani LM, Liberman MY, Malhotra AK, Simpson W, Kane JM (2025) Automated speech and language markers of longitudinal changes in psychosis symptoms. NPP Digit Psychiatry Neurosci 3:13. 10.1038/s44277-025-00034-z PMC12173942 40538505

[67] Yu Z, Guindani M, Grieco SF, Chen L, Holmes TC, Xu X (2022) Beyond t test and ANOVA: applications of mixed-effects models for more rigorous statistical analysis in neuroscience research. Neuron 110:21–35. 10.1016/j.neuron.2021.10.030 PMC8763600 34784504

