# Supplementary material for: Sex-specific clinical profiles and long-term outcomes in adolescents and young people at clinical high risk for psychosis: Findings from the PARMS program
Source: Eur Child Adolesc Psychiatry. 2026 Jun 4;35(10):3129–43. doi: 10.1007/s00787-026-03082-8 (PMC13696591; doi:10.1007/s00787-026-03082-8)
Supplement: Supplementary file 1 [file 787_2026_3082_MOESM1_ESM.docx]

Table S1. The 8 PANSS items indicated by the “Remission in Schizophrenia Working Group’s criteria” to define symptomatic remission.

P1: Delusions

P3: Hallucinatory behavior

G9: Unusual thought content

P2: Conceptual disorganization

G5: Mannerisms and posturing

N1: Blunted affect

N4: Passive/apathetic social withdrawal

N6: Lack of spontaneity and flow of conversation

Scores of ≤3 on these 8 items must be maintained for a period of 6 months.

Note. PANSS = Positive And Negative Syndrome Scale.

Table S2 – Specialist psychopharmacological and psychosocial treatment package of the PARMS program.

As recommended by current EIP official guidelines (Schmidt et al., 2015; RER, 2023) *Antipsychotic* (AP) medication should be prescribed to CHR-P subjects who had (a) a rapid decline in daily functioning, (b) a sudden escalation of overt psychotic symptoms, (c) an immediate risk of suicide or severe violence, or (d) who failed to respond to psychosocial interventions. As first-line pharmacological treatment, low-dose atypical AP drugs were used (Poletti et al., 2021).
*Individual psychotherapy* was shaped on the model by van der Gaag and his team for young people at CHR-P, offering at least 15 sessions per year (each lasting 60 minutes) (van der Gaag et al., 2012). *Family psychoeducation* was adapted on the model by McFarlane for CHR-P individuals, providing at least 10-12 sessions to each family (McFarlane et al., 2016). Finally, a dedicated *case-manager* was also provided to each subject/family to coordinate all interventions planned, especially those aimed at promoting a recovery-oriented early rehabilitation through at least 24 sessions provided along the 2 years of follow-up (each lasting 60 minutes) (Ficarelli et al., 2021).

Note. PARMS = Parma At-Risk Mental States.

Schmidt, S.J., Schultze-Lutter, F., Schimmelmann, B.G., Maric, N.P., Salokangas, R.K., Riecher-Rössler, A., van der Gaag, M., Meneghelli, A., Nordentoft, M., Marshall, M., Morrison, A., Raballo, A., Klosterkötter, J., Ruhrmann, S. (2015). EPA guidance on the early intervention in clinical high risk states of psychoses. European Psychiatry, 30, 388-404. <https://doi.org/10.1016/j.eurpsy.2015.01.013>.

Regione Emilia-Romagna (RER). (2023). Raccomandazioni regionali per la promozione della salute e del benessere in persone all’esordio psicotico, seconda edizione. Bologna: Centro Stampa della Regione Emilia-Romagna.

van der Gaag, M., Nieman, D.H., Rietdijk, J., Dragt, S., Ising, H.K., Klaassen, R.M., Koeter, M., Cuijpers, P., Wunderink, L., Linszen, D.H. (2012). Cognitive behavioral therapy for subjects at ultra-high risk for developing psychosis: a randomized controlled clinical trial. Schizophrenia Bulletin, 38, 1180-1188.<https://doi.org/10.1093/schbul/sbs105>.

McFarlane, W.R. (2016). Family interventions for schizophrenia and the psychoses: a review. Family Process, 55, 460-482. <https://doi.org/10.1111/famp.12235>.

Ficarelli, M.L., Troisi, E., Vignali, E., Artoni, S., Franzini, M.C., Montanaro, S., Andreoli, M.V., Marangoni, S., Ciampà, E., Erlicher, D., Pupo, S., Pelizza, L. (2021). Implementing individual and placement support for patients with severe mental illness: findings from the real world. Journal of Psychopathology, 27, 71-80. https://old.jpsychopathol.it/wp-content/uploads/2021/06/01_346_Ficarelli-1.pdf.

Figure S1 – Mixed design ANOVA results: between-group comparisons on profile plots of statistically significant clinical and outcome parameters across the 2-year follow-up period in the two CHR-P subgroups.

**SOFAS scores**

Note. ANOVA = Analysis of Variance; CHR-P = Clinical High Risk for Psychosis; SOFAS = Social and Occupational Functioning Assessment Scale; HoNOS = Health of the Nation Outcome Scale; PANSS = Positive And Negative Syndrome Scale; T0 = baseline assessment time; T1 = 1-year assessment time; T2 = 2-year assessment time. Only CHR-P participants who concluded the 2-year follow-up period were included in the analysis.

Figure S2. Mixed design ANOVA results: Q-Q plots (for graphic analyses of residuals) related to clinical and outcome parameters resulted statistically different between the two CHR-P subgroups across the 2-year follow-up period.

Q-Q Plot: PANSS “Negative symptoms” factor scores

Q-Q Plot: PANSS G12 “Lack of judgement/insight” item scores.

Q-Q Plot: SOFAS scores

Q-Q Plot: HoNOS “Social problems” domain scores

Note. PANSS = Positive And Negative Syndrome Scale; SOFAS = Social and Occupational Functioning Assessment Scale; HoNOS = Health of the Nation Outcome Scale.

Table S3 – Mixed-design ANOVA results: clinical outcome parameters across the 1-year follow-up period in the two CHR-P subgroups.

| Variable | Time effects | | | | | | Group effects | | | | | Interaction effects  (time x group) | | | |
| --- | --- | --- | --- | --- | --- | --- | --- | --- | --- | --- | --- | --- | --- | --- | --- |
|  | df | F | | p | η2 | | df | F | p | | η2 | df | F | p | η2 |
| *PANSS scores*  Positive symptoms  Negative symptoms  Disorganization  Affect  Resistance/Excitement-activity  Total score  G12 Lack of judgment/insight item^*^  SOFAS score  *HoNOS scores*  Behavioral problems  Impairment  Psychiatric symptoms  Social problems  Total score | 1  1  1  1  1  1  1  1  1  1  1  1  1 | 8.622  67.381  12.479  65.713  .190  52.587  .964  156.404  77.321  55.081  158.147  111.837  185.745 | | **.004**  **.001**  **.001**  **.001**  .663  **.001**  .328  **.001**  **.001**  **.001**  **.001**  **.001**  **.001** | .067  .359  .093  .352  .002  .305  .008  .535  .320  .251  .491  .405  .531 | | 1  1  1  1  1  1  1  1  1  1  1  1  1 | 1.129  5.995  .219  2.215  1.660  1.695  5.835  4.121  .464  .961  .535  3.628  .924 | .290  **.016**  .641  .139  .560  .195  **.017**  **.042**  .497  .328  .466  **.049**  .338 | | .009  .048  .002  .018  .003  .014  .046  .031  .003  .006  .003  .029  .006 | 1  1  1  1  1  1  1  1  1  1  1  1  1 | 1.650  1.158  2.056  .039  .341  1.381  .287  1.033  .197  .719  1.978  .719  1.073 | .201  .284  .154  .843  .560  .242  .593  .311  .657  .398  .162  .398  .302 | .013  .010  .017  .001  .003  .011  .002  .008  .001  .008  .012  .004  .006 |
| Variable  (Group effects) | | | EMM (SE) | | | | | | |  |  |  |  |  |  |
|  |  |  | Males  (n=85) | | | Females  (n=89) | | | |  |  |  |  |  |  |
| T0 PANSS Negative symptoms  T1 PANSS Negative symptoms | | | 21.340 (1.026)  16.415 (.999) | | | 17.739 (.899)  13.957 (.875) | | | |  |  |  |  |  |  |
| T0 G12 PANSS “Lack of Judgment/Insight  T1 G12 PANSS “Lack of Judgment/Insight | | | 2.593 (.187)  2.444 (.195) | | | 1.971 (.166)  1.928 (.172) | | | |  |  |  |  |  |  |
| T0 SOFAS score  T1 SOFAS score | | | 47.339 (1.076)  57.613 (1.655) | | | 49.789 (.972)  61.882 (1.495) | | | |  |  |  |  |  |  |
| T0 HoNOS Social problems  T1 HoNOS Social problems | | | 6.568 (.458)  4.815 (.393) | | | 5.776 (.344)  3.718 (.272) | | | |  |  |  |  |  |  |

Note. ANOVA = analysis of variance; CHR-P = Clinical High Risk for Psychosis; HoNOS = Health of the Nation Outcome Scale; PANSS = Positive And Negative Syndrome Scale; SOFAS = Social and Occupational Functioning Assessment Scale; df = degrees of freedom; F = F statistic value; p = statistical significance; η2 = partial eta squared; EMM = Estimated Marginal Mean; SE = Standard Error. As all Mauchly’s tests of sphericity are statistically significant (p<0.05), Greenhouse–Geisser corrected degrees of freedom to assess the significance of the corresponding F value are used. Statistically significant p values are in bold. Bonferroni’s corrected p values are reported. Only FEP participants who concluded the 2-year follow-up period are included in the analysis. ^*^The PANSS “Lack of judgment/insight” item is emphasized because it is not part of the five-factor model proposed by Shafer and Dazzi (2019).

Table S4. Binary logistic regression results for 2-year HoNOS functional remission: multivariate analysis.

| Dependent variable  (2-year HoNOS functional remission) | Estimate | SE | HR | p | 95% CI for HR  Lower Upper | |
| --- | --- | --- | --- | --- | --- | --- |
| Intercept  Gender (males)  Age (at entry)  NEET status  Substance misuse (at entry)  PANSS total score | -1.908  -1.271  .073  -.059  .593  .658 | 2.518  .526  .070  .028  .658  .516 | .148  .281  1.075  .943  1.810  1.930 | .449  **.046^*^**  .299  .065  .367  .203 | -6.843  -2.301  -.064  -.114  -.697  -.345 | 3.027  -.204  .210  .004  1.883  1.669 |

Note. HoNOS = Health of the Nation Outcome Scale; NEET = Not [engaged] in Education, Employment or Training; PANSS = Positive And Negative Syndrome Scale; SE = Standard Error; HR = Hazard Ratio; 95% CI = 95% confidence intervals for HR; p = statistical significance. ^*^Bonferroni corrected p values are reported. Significant statistical p values are bold. Only CHR-P participants who concluded the 2-year follow-up period are included in the analysis. HoNOS functional remission = HoNOS item 9, 10 and 11 subscores <2.

| Table S5. Attrition analysis results in the CHR-P total sample (n=179)Baseline variable | Dropped out participants  (n=27) | Not dropped out participants  (n=152) | X^2^/U | p |
| --- | --- | --- | --- | --- |
| Sex (males)  Age (at entry)  Education (in years)  DUI (in weeks)  *PANSS scores*  Positive symptoms  Negative symptoms  Disorganization  Affect  Resistance/Excitement-activity  Total score  SOFAS score | 13 (48.1%)  19.07±3.65  10.67±1.86  35.85±34.78  10.50±3.50  17.18±6.62  14.27±5.17  13.18±5.09  8.04±3.59  65.41±17.43  50.74±9.59 | 76 (50.0%)  19.60±3.83  11.46±2.51  48.47±50.82  10.81±3.84  19.77±7.81  15.96±5.40  15.77±5.07  7.08±3.12  71.81±17.25  48.25±8.35 | .031  1894.500  1647.50  1804.000  1094.000  894.000  861.000  801.500  1252.500  882.000  2489.000 | .859  .525  .098  .318  .913  .171  .099  .061  .306  .147  .085 |

Note. CHR-P = Clinical High Risk for Psychosis; DUI = Duration of Untreated Illness; PANSS = Positive And Negative Syndrome Scale; SOFAS = Social and Occupational Functioning Assessment Scale. Frequencies (and percentages), mean ± standard deviation, Chi-square (X^2^) and Mann-Whitney (U) test values are reported. Statistically significant p values are in bold.
